# A novel approach to screening and managing the urinary tract infections suspected sample in the general human population

**DOI:** 10.3389/fcimb.2022.915288

**Published:** 2022-08-25

**Authors:** Magdalena Szmulik, Zuzanna Trześniewska-Ofiara, Mariola Mendrycka, Agnieszka Woźniak-Kosek

**Affiliations:** ^1^ Sysmex Poland Ltd, Scientific Aspect Prepared in Cooperation with Department of Laboratory Diagnostics, Military Institute of Medicine, Warsaw, Poland; ^2^ Department of Laboratory Diagnostics, Mazovian Specialist Hospital Ltd, Radom, Poland; ^3^ Department of Nursing, Faculty of Medical Sciences and Health Sciences, Kazimierz Pulaski University of Technology and Humanities, Radom, Poland; ^4^ Department of Laboratory Diagnostics, Military Institute of Medicine, Warsaw, Poland

**Keywords:** urinary tract infections, antimicrobial resistance, urinalysis, ruling out UTI in general practice, UTI diagnostic approach

## Abstract

**Background:**

Automated urine technology providing standard urinalysis data can be used to support clinicians in screening and managing a UTI-suspected sample. Fully automated urinalysis systems have expanded in laboratory practice. Commonly used were devices based on digital imaging with automatic particle recognition, which expresses urinary sediment results on an ordinal scale. There were introduced fluorescent flow cytometry analyzers reporting all parameters quantitatively. There is a need to harmonize the result and support comparing bacteria and WBC qualitative versus semiquantitative results.

**Methods:**

A total of 1,131 urine samples were analyzed on both automated urinalysis systems. The chemical components of urinalysis (leukocyte esterase and nitrate reductase) and the sediment results (leukocytes and bacteria) were investigated as potential UTI indicators. Additionally, 106 specimens were analyzed on UF-5000 and compared with culture plating to establish cut-offs that can be suitable for standard urinalysis requirements and help to guide on how to interpret urinalysis results in the context of cultivation reflex.

**Results:**

The medians of bacteria counts varies from 16.2 (absence), 43.0 (trace), 443.5 (few), 5,389.2 (moderate), 19,356.6 (many) to 32,545.2 (massive) for particular digital microscopic bacteriuria thresholds. For pyuria thresholds, the medians of WBC counts varies from 0.8 (absence), 2.0 (0-1), 7.7 (2-3), 21.3 (4-6), 38.9 (7-10), 61.3 (11-15) to 242.2 (>30). Comparing the culture and FFC data (bacterial and/or WBC counts) was performed. Satisfactory sensitivity (100%), specificity (83.7%), negative predictive value (100%), and positive predictive value (75%) were obtained using indicators with the following cut-off values: leukocytes ≥40/µl or bacteria ≥300/µl.

**Conclusions:**

Accurate urinalysis gives information about the count of bacteria and leukocytes as useful indicators in UTIs, in general practice it can be a future tool to cross-link clinical and microbiology laboratories. However, the cut-off adjustments require individual optimization.

## Introduction

Urinary tract infections (UTIs) are a severe public health problem. UTIs are some of the most common bacterial infections and the most frequent healthcare-associated infection (nosocomial as well as post-hospital infections) (Bermingham and Ashe, 2012; Foxman, 2014; Wagenlehner et al., 2020). Concerning the prevention, diagnostics, treatments, and monitoring of urinary tract infections, it is important to address the urgent aspects of antimicrobial stewardship. UTIs, in terms of global deaths associated with and attributable to resistance in lower respiratory infections, bloodstream infections, and intra-abdominal infections, are the most common infections in humans (Gágyor et al., 2021; Murray et al., 2022). The impact of antimicrobial resistance (AMR) estimated as deaths per year is projected as even higher than cancer disorders and increases the general healthcare costs considerably. Drug prescription based on the symptoms is performed commonly, but inappropriate antimicrobial treatment is one of the reasons for antimicrobial resistance to develop (Mendelson and Matsoso, 2015; Dadgostar, 2019; Jiang and Chen, 2020). The antibiotics for suspected UTI infections consist of approximately 15.0% of all prescribed antibacterial drugs. The ineffectuality of regularly prescribed antibiotics is estimated between 8 and 65% of UTI cases (Costelloe et al., 2010; Poole et al., 2020; Heinz et al., 2020).

There are heterogeneous clinical phenotypes of UTIs; therefore, different classification and diagnostic algorithms for UTIs exist. The European Association of Urology proposed the most widely used UTI classification and recommends diagnostic laboratory tests to recognize UTIs in particular cases: including test strips [detection of leukocyte esterase (LE) activity and nitrite positivity (NIT)] and culture regarding cultivation; significant bacteriuria is the laboratory indicator that permits distinguishing of UTI and contamination. Different definitions of significant bacteriuria exist, which is defined as presence from 10^5^, 10^4^, to 10^3^ CFU/ml (European Confederation of Laboratory Medicine, 2000; Bonkat et al., 2021). Both methods are impacted by well-known limitations (NIT/LE: multiple reasons for false-positive and false-negative results, too low sensitivity and specificity for being the only screening parameters for bacteriuria; cultivations: long turnaround time, high ratio of negative results, and high costs). In general practice and local guidance results for leukocytes and bacteria assessed from urine, sediments play a role to screen the urine sample. The LET-NPT and WBC combined and/or BACT present in urine particle analysis has increased the sensitivity of UTI diagnosis (Cortes-Penfield et al., 2017; Radmayr et al., 2018; Bil-Lula et al., 2019; Bhugra and Gachinmath, 2021). For higher impact, standardization such as WBC and RBC, given as counts per litre/microlitre instead of counts per high power field, is required. Although during the last decades more automated systems for urinalysis have become available to report results quantitatively, expressing urinary sediment results on an ordinal scale is still common in Poland. Physicians and clinical laboratories requested doubts when comparing the results obtained by different methods. This study was designed to compare the laboratory indicators of UTIs presenting both resulting report forms as counts per litre/microlitre and counts per high power field. We aimed to evaluate the clinical usefulness of UF-4000 in the microbiology context and assess the potential strategy to help in the interpretation of the qualitative results and in the management of further examination of patients suspected of UTIs.

## Materials and methods

### Study population and clinical samples

Samples from the Military Institute of Medicine in Warsaw (MIM) patients who attended the Central Laboratory between September 2018 and October 2019 were randomly selected. For the first part of the study, exactly 1,131 freshly collected urine specimens were eligible for further examinations. For the second part of the study, 106 samples requested for routine microbiology diagnostic purposes were examined. Urine samples with inadequate volume for analysis were excluded. The urine specimens were collected into sterile containers/tubes without any preservatives. The study was approved by the Ethics Committee of MIM with protocol number 17/WIM/2019.

### Urine sample processing

Requested automated urinalysis was performed firstly with a routine-work analyzer (Iris iQ^®^200 ELITE/iChem VELOCITY, Beckman Coulter, CA, USA). The measurement includes test strips and digital images for each sample. During the study period, the laboratory presented the results of sediment examination in units per high power field (HPF) for WBC and RBC, and bacteria were reported semi-quantitatively (on a scale from 0 to 5). From a technical point of view, the analyzer can obtain RBC, WBC, and EC results and present them as units of cells per milliliter, but the units of cells per field were well established among clinicians. All samples derived from the Central Laboratory were tested by the UF-4000 analyzer (Sysmex Corporation, Kobe, Japan) within 30 min. During the study period, all urine particle results based on UF-4000 were reported per microliter. The cultures were performed in the Microbiology Laboratory of MIM. All these processes were carried out within 2hrs of sample reception. All analyzers measured native urine specimens.

### Automated urine microscopic analysis

Until now, there is no established reference method for urinalysis. Automatic particle recognition was applied in routine clinical laboratories in the past few years. This technique replaced traditional urinary sediment examination because of carrying out automated urinalysis with sufficient correlations with manual microscopic urinary sediment examination (Oyaert and Delanghe, 2019; Queremel Milani and Jialal, 2022). The iQ200 Elite (International Remote Imaging Systems) analyzer is an automated urine microscopy analyzer based on flow imaging technology and auto-particle recognition located at the view station—a personal computer equipped with automated particle recognition software. The measurement technique is supported by hydrodynamically oriented flow, which means that the urine particles are suspended and further sent directly by a constant flow of sheath fluid, which guarantees that cells will be positioned, one by one, within the focal plane on a microscope objective and will be photographed. The software is based on size, shape, texture, and contrast features that categorize urine particles into 12 dedicated groups, including RBC, WBC, WBC clumps, hyaline casts, unclassified casts, EC, non-squamous ECs, bacteria, yeast, crystals, mucus, sperm, amorphous substances, and “all small particles” (ASP). This group consists of unclassified urine particles of <3 μm. This category can include bacteria (such as cocci crystals and other formed elements) and should be reviewed by a trained technician to be placed into the right category if necessary (Shayanfar et al., 2007; Mayo et al., 2008; Foudraine et al., 2018).

### Fluorescent flow cytometric analysis

Urinalysis flow cytometer technique has been used routinely to replace the traditional sediment urinalysis for almost two decades (Cho et al., 2018). The manufacturer recently introduced UF-5000/4000 as fully automated analyzers that count and classify the major urine particles (cells: white blood cells, red blood cells, bacteria, epithelial cells with detailed differentiation, yeast-like cells, and crystals). Additionally, Sysmex UF-series analyzers have UTI screening capabilities proved by numerous studies that observed WBC and bacteria amount as UTI indicators (Shang et al., 2013). A quantitation and differentiation principle is based on the measurement of the signals which were generated by the particles suspended in a fluid stream and passed through detection systems after a laser light shines on particles and cells. The detected signals are shown as dots on the scattergrams, which is a useful tool to classify and describe the morphology and characteristics of urine particles. This technology enables the provision of detailed characteristics of the particle, such as information about the identification of bacterial Gram stainability (BACT-info). The final classification is grounded on the various types of light intensity emitted by each particle. Signals express the particular features of cells or formed elements in urine. Forward scattered light corresponds to the size, shape, and volume, while fluorescence light is detected in cells containing genetic materials (nucleic acids) (Previtali et al., 2017).

### Statistical analysis

Anonymized study data were entered into a Microsoft Excel spreadsheet. To define the sample values and compare the WBC and BACT results from the three devices, descriptive statistics were used. The types of distribution of leukocyte and bacteria results were verified using the Shapiro–Wilk test. The results were analyzed using descriptive statistics and the Kruskal–Wallis test with *post-hoc* Dunn’s test for nonparametric data. Differences between the bacterial and leukocyte positive rates of the patient’s results were compared using the chi-square test. The *p*-value considered statistically significant was 0.05. The analysis comparing the UF-4000 and culture results was performed by making crosstabs for the confusion matrix. Statistical analysis was carried out using Microsoft Excel (Windows, Microsoft Office Professional, 2016) and IBM SPSS Statistics 25 software version (IBM Corp.).

## Results

### Demographic data

In total, 1,131 urine samples were analyzed from 22 MIM hospital departments that requested urinalysis tests, including the emergency department (*n* = 60; 5.48%). The 10 most frequently requested departments are shown in **Table 1**. Furthermore, 50% of all examined samples have come from the first six listed departments. The remaining departments hold about 4 to 5% stake in all requested samples.

**Table 1:** Number and percent values of patients according to the departments that requested urinalysis.

Clinic/department	*N*	%
Clinical Department of Pediatrics and Pediatrics Nephrology and Allergology	131	11.58
Clinical Department of Internal Medicine and Rheumatology	120	10.61
Clinical Department of Internal Medicine, Nephrology and Dialysis Therapy	115	10.17
Clinical Oncology Department	81	7.16
Clinical Neurology Department	76	6.72
Clinical Department of Cardiology and Internal Medicine	70	6.19
Clinical Department of Internal Medicine and Hematology	66	5.84
Department of Trauma and Orthopedics	66	5.84
Emergency Department	62	5.48
Department of Gastroenterology, Internal Medicine and Endocrinology	52	4.60
Others	292	25.81

Demographic information such as gender and age are presented in **Table 2**. Samples from females were more prevalent than from male patients. A significant peak was observed on a distribution of samples based on age; it was in a range between 68 and 70 years.

**Table 2:** Demographic information of the study group.

Patients	*N*	%	Min	Max	Median
Female	680	60.1	1	97	57
Male	451	39.9	1	94	57
Total	1,131	100.0	1	97	57

Pearson correlation tests were performed for age, and results were obtained from all three methods—urine dipstick, image-based microscopy, and flow cytometry—which described WBC and bacteria. For all parameters and methods, there were positive, weak correlations. With age, an increased presence of urinary particles (WBC and BACT) was observed. The strongest correlation in this group that we observed was between age and the count of bacteria measured by fluorescent flow cytometry (FFC) (Pearson’s *r* = 0.13; *p* < 0.001) compared with digital microscopy (Pearson’s *r* = 0.11; *p* < 0.001), and urine dipstick (Pearson’s *r* = 0.09; *p* < 0.002). In contrast, the highest correlation coefficient was observed in the WBC group as measured by automated microscopy (Pearson’s *r* = 0.21; *p* < 0.001) compared with WBC from FFC (Pearson’s *r* = 0.09; *p* < 0.004) and urine dipstick (Pearson’s *r* = 0.18; *p* < 0.004).

### Method comparison

We decided to base the definition of positive or negative value for particular parameters on appropriate thresholds. This study focused on WBC and BACT analytical performance according to their cut-off thresholds for each technique. For the digital-based way, it was the previously established and routinely used reference ranges, and for FFC it was literature and manufacturer data for adults (cut-off value for BACT: 300 cells/µl and WBC: 25 cells/µl). The digital image method was adjusted to describe bacteria in a semiquantitative way, but FFC reports BACT results as a count of bacteria in a microliter and, additionally, can deliver information about the identification of bacterial Gram stainability (BACT-info). Urine LE nitrate and automated microscopy WBC and bacteria were analyzed using conventional assessment values (negative, trace, 1+, 2+, and 3+) and then dichotomized into positive or negative (see **Table 3**) with trace values considered as positive. Regarding urine leukocytes, specimens with some cells higher than WBC of 0–10 cells/HPF were categorized as negative, and all other values were categorized as positive. All possible test results from each method are shown in **Table 3**.

**Table 3:** Possible test results of three methods.

Test method	Parameter	Count	Value (Positive /Negative)
**Urine dipstick**	**Nitrate**	**-**	**Negative**
		**0.1 mg/dl**	**Positive**
		**0.2 mg/dl**	**Positive**
	**Leukocyte esterase**	**-**	**Negative**
		**25/µl**	**Positive (+)**
		**75/µl**	**Positive (++)**
		**250/µl**	**Positive (+++)**
		**500/µl**	**Positive (++++)**
**Urine sediment based on the digital method**	**Bacteria**	**Absence**	**Negative**
		**Trace**	**Negative**
		**Few**	**Positive (+)**
		**Moderate**	**Positive (++)**
		**Many**	**Positive (+++)**
		**massive**	**Positive (++++)**
	**Leukocytes**		
		**0/HPF**	**Negative**
		**1/HPF**	**Negative**
		**2-3/HPF**	**Negative**
		**4-6/HPF**	**Negative**
		**7-10/HPF**	**Negative**
		**11-15/HPF**	**Positive (+)**
		**16-20/HPF**	**Positive (++)**
		**21-30/HPF**	**Positive (+++)**
		**>30/HPF**	**Positive (++++)**
		**>60/HPF**	**Positive (+++++)**
		**>100/HPF**	**Positive (++++++)**
**Flow cytometry**	**Bacteria**	**<300/µl**	**Negative**
		**≥300/µl**	**Positive**
		**≥100 000/µl**	**Positive (++++)**
	**Leukocytes**	**<25/µl**	**Negative**
		**≥25/µl**	**Positive**

### Comparison of flow cytometric *versus* automated microscopy leukocytes and bacteria results

Flow cytometry measurements generated the highest percentage of positive results for bacteria parameters (**Table 4**). There was a difference between FFC and automated microscopy measurements in the frequency of positive WBC urine test [*χ* ^2^(2) = 17.68; *p* < 0.001].

**Table 4:** Urine positive test results in the different methods of urine measurement.

Test strips			Digital imaging			Flow cytometry		
	*n*	%		*n*	%		*N*	%
Leukocyte esterase	370	32.7	WBC	261	23.1%	WBC	349	30.9
Nitrate	103	9.1	Bacteria	308	27.2	Bacteria	360	31.9

We studied the relationship between the microscopic bacteriuria and pyuria thresholds described in routine laboratory examination and flow cytometry values observed in these groups (see **Tables 5**, **6**). The Kruskal–Wallis test was performed for bacteria (*p* < 0.001) and leukocytes (*p* < 0.001). All differences were statistically significant, and the effect size was relatively strong. Additionally, Dunn’s test was performed to pinpoint which specific means are significantly different from the others. More precisely, for bacteria, the effect size was significantly strong for a combination of group pairs: absence *vs.* trace, absence *vs.* few, and trace vs few. In detail, for WBC, there were statistically significant differences in only one threshold: two to three leukocytes/HPF. In the digital-based result group (from 1+ to 4+), the value of the median increased sequentially. Among the 289 patients with bacteriuria ≥1+, a median of the flow cytometry results is higher than 300 cells/µl (see **Table 5**).

**Table 5:** Test characteristics of the two automated systems used to describe bacteriuria.

	Digital imaging thresholds	Interpretation	*N*	Median	IQR	*p*
BACT (UF-4000)	Absence	**Negative**	329	16.2	50.0	<0.001
	Trace	**Negative**	493	43.0	201.7	
	Few	**Positive (+)**	140	435.5	2,415.0	
	Moderate	**Positive (++)**	73	5,389.2	43,146.7	
	Many	**Positive (+++)**	36	19,356.6	49,741.5	
	Massive	**Positive (++++)**	40	32,545.2	45,473.6	

**Table 6:** Test characteristics of the two automated systems to describe pyuria.

	Digital imaging thresholds	*N*	Median	IQR	*p*
WBC (UF-4000)	Absence	40	0.8	0.9	<0.001
	0 to 1	449	2.0	2.3	
	2 to 3	222	7.7	9.0	
	4 to 6	99	21.3	18.3	
	7 to 10	60	38.9	32.0	
	11 to 15	55	61.3	33.2	
	16 to 20	38	96.4	57.3	
	21 to 30	32	147.9	100.7	
	>30	55	242.2	179.2	
	>60	21	419.9	2,77.8	
	>100	60	1,377.4	2,753.1	

### Comparison of flow cytometric results *versus* culture

Among 106 urine cultures, 46 (43.4%) were positive and 60 were negative (56.6%). Of the 46 bacteria-positive urine cultures, 27 (25.5%) were of significant growth and 19 were of nonsignificant growth (mixed flora, 17.9%). Of the 27 positive cultures, 24 were positive at 10^5^ CFU/ml, two were positive at 10^4^ CFU/ml, and one was positive at 10^3^ CFU/ml. We identified in the 27 positive cultures a total of seven species. In descending order of prevalence, these were *Escherichia coli* (*n* = 14), *Enterococcus faecalis* (*n* = 4), *Pseudomonas aeruginosa* (*n* = 4), *Klebsiella pneumoniae* (*n* = 3), *Proteus mirabilis* (*n* = 3), *Klebsiella oxytoca* (*n* = 2), and *Enterococcus faecium* (*n* = 1). Thus, four samples had two species.

**Table 7** presents the distribution of the culture results regarding the examination cut-offs. We evaluated two models for predicted positive culture (10^5^ CFU/ml) and ruled out negative samples. In the two models, we used both bacteria and WBC (combined or separately). Two values for WBC (25/µl and 40/µl) and one for BACT were evaluated. The first one is based on the interchangeableness and needs only one raised parameter. The second algorithm was adjusted to check if both combined parameters were above the cut-offs. A comparison of performance SE and SP of these two models shows that the first algorithm was higher SE (100%) than bacteria and WBC considered together (79.2 and 70.8%). The second model allows a higher ratio of culture reduction to be obtained.

**Table 7:** Evaluation of different cut-off points and parameters combinations to predict positive urine culture (*adopted in this study cut-offs).

	WBC or BACT			WBC or BACT			WBC and BACT			WBC and BACT		
	Total	M	K	Total	M	K	Total	M	K	Total	M	K
Cut-off—WBC	25	25	25	40*	40	40	25	25	25	40	40	40
Cut-off—BACT	300	300	300	300*	300	300	300	300	300	300	300	300
Total samples	106	57	48	**106**	57	48	106	57	48	106	57	48
Positive samples	33	19	14	**32**	18	14	20	12	8	18	12	6
Negative samples	40	25	14	**41**	26	14	53	32	20	55	32	22
Contaminated samples	33	13	20	**33**	13	20	33	13	20	33	13	20
Sensitivity (%)	100	100	100	**100**	100	100	79.2	84.6	72.7	70.8	84.6	54.5
Specificity (%)	81.6	80.6	82.4	**83**.**7**	83.9	82.4	98	96.8	100	98.0	96.8	100
NPV (%)	100	100	100	**100**	100	100	90.6	93.8	85.0	87.3	93.8	77.3
PPV (%)	72.2	68.4	78.6	**75**	72.2	78.6	95.0	91.7	100	94.4	91.7	100
False negative rate (%)	0	0	0	**0**	0	0	4.7	3.5	6.3	6.6	3.5	10.4
Culture reduction (%)	–	43.9	29.2	**-**	45.6	29.2		56.1	41.7		56.1	45.8
	37.7	37.1		**38**.**7**	52.4		50.0	49.5		51.9	51.4	

## Discussion

The laboratory diagnostic approach of UTI plays an important role in the general healthcare system. It supports clinicians to make therapeutic decisions, monitoring not only renal and urinary tract diseases, has a direct impact on decreasing the general cost of healthcare from a long-term perspective, and can avoid the spread of antimicrobial resistance (Cavanaugh and Perazella, 2019; Queremel Milani and Jialal, 2022). International, national, and/or local guidelines provide the diagnostic algorithms to diagnose UTIs. These guidelines frequently use the concept of uncomplicated and complicated UTIs and treat test strip examination as a first-choice test, which can or should be in the case of a high category of risk factors, further causing reflex microbiology examinations, respectively (Żurowska et al., 2016; Hryniewicz et al., 2017; Radmayr et al., 2022). In the UTI and AMR context, prioritization of progress focused on finding UTI markers and enabling easy, cheap, and quick screening of a large number of the population during regular medical practice seems to be necessary. Ground-breaking technical aspects and workflow innovations on the market are promising in this direction (Pezzlo, 2014a; Foudraine et al., 2018).

The role of sediment results seems to be underestimated until now, probably because of the lack of standardization for manual methods and the poor repeatability of results (Bednarczuk et al., 2011; Bil-Lula et al., 2019; Queremel Milani and Jialal, 2022). Due to the long tradition of using manual microscopy in Polish laboratories, it is the most popular urinalysis method. According to the frequency in the EQA program (organized by Labquality, 2017–2019, Helsinki, Finland), this was equal to 63% of all surveys. There are still laboratories using the coverslip method, but automated urinalysis becomes more common. There were 35% of automated measurements, with 30% performed by image-based technology and 5% by fluorescent flow cytometry. The Polish guidelines recommend that at least WBC and RBC results should be expressed as the number of particles × 10^6^/L, but the manual method still holds qualitative description for bacteria in manual examinations, including five categories (Ćwiklińska et al., 2020). When the quantification values for bacteria are commonly attributed to microbiology investigations, the expressed bacterial count as the number of particles × 10^6^/L can be confusing. The comparison of urine WBC and BACT counts by urine flow cytometry to conventional reports is the first study in Poland. This data can support physicians with the daily routine of diagnosis and control of UTIs, especially in cases based on the mixed form of results. It can enable the laboratory staff to be proficient in the comparison of bacteria and WBC qualitative *versus* semiquantitative results.

To avoid prematurely mislabeling a patient with a diagnosis of UTI by laboratory processing and reporting of urinalyses, a microbiology pilot study, comparing UF-4000 results with culture plating, was performed. We found it crucial to establish cut-offs that can be suitable for standard urinalysis requirements and help to guide on how to interpret urinalysis results in the context of cultivation reflex testing.

The features of the studied method can also be crucial information for diagnostic purposes in microbiology labs (Kim et al., 2021). The presence or absence of inflammation in the urinary tract can be predicted by bacteria and white blood cells. Accurate urinalysis that can give information about the count of bacteria and leukocytes as useful indicators in UTI is a future tool to cross-link clinical and microbiology laboratories (Gilboe et al., 2021). Oyaert et al. recommended incorporating pyuria as an indicator for UTI microbiological diagnosis. This publication marked the definition of pyuria and categorized it by age and assessment technique. The ranges for flow cytometry technique are as follows: adults ≥20–25 WBC/µl and pediatrics (0–2 years of age) ≥10–25 WBC/µl. Oyaert et al. proposed a cut-off ≥10 WBC/µl for all patients examined by the microscopic technique (Williams et al., 2010; Oyaert et al., 2018). Regarding the pyuria assessment by urine dipstick, a higher percentage for positive LE frequency can be brought about by the presence of lysed WBC and urine dipstick capability to measure leukocyte enzyme released from cells and leukocytes that also remained intact. It should be noted that the test strip examination was performed as a first measurement; so, in comparison to the rest of the positive results, the impact of time between collection and measurement is not significant. The dipstick tests are vulnerable to interferences and method limitations. The nitrate reductase detection tests can be affected and lead to false-negative results. Pre-analytical phase aspects such as short incubation time of urine in the bladder and short supply of dietary nitrate intake can determine the results. This limitation hit principally pediatric patients and patients with polyuria as well. Additionally, test pads for NIT are sensitive only to bacteria that can convert nitrate to nitrite (Williams et al., 2010). There are more reasons for the false-negative test for nitrate reductase, and it can be independent of test manufacture (Kavuru et al., 2020). In summary, the differences between low bacteria positive frequency in dipstick test *versus* image recognition and flow cytometry techniques are probably explained by the test strip method principle and limitation and obvious interferences (Pieretti et al., 2010; Pezzlo, 2014b; Mambatta et al., 2015; Riley and McPherson, 2017).

Possible associations between the urine culture and the results of urinalysis obtained by the different methods were investigated by numerous researchers. To rule out negative samples and predict positive cultures, various techniques are evaluated: urine dipstick (LE and NIT presence), urinalysis methods (bacteriuria/pyuria categorical microscopic thresholds or exact cell counts), and complex algorithms including patients’ data and UTI risk factors (Williams et al., 2010).

Several studies compared the UF-5000/4000 results with urine cultivation results. The design of the studies indicated urine flow cytometry screening for testing the pyuria and/or bacteriuria before culture plating. The authors stated that flow cytometry has a good sensitivity to predict significant bacteriuria. Studies differ by group size, criteria of UTI suspicion, definitions of significant bacteriuria, and adopted cut-off values.

Haugum et al. tested WBC and BACT alone or in combination and took into account, gender differences. The cut-offs for bacteria and WBC were adjusted to 30/μl for both parameters, while significant bacteriuria was defined as 10^4^ with sensitivity of 96.6%, specificity of 55.5%, negative predictive value (NPV) of 92.3%, and positive predictive value (PPV) of 74.7%, which were slightly lower than our results (sensitivity, 100%; specificity, 83.7%; NPV, 100%; and PPV, 75%). In their study, the negative predictive value was lower when WBC and BACT were tested alone (69.9% and 91.2%, respectively). In the total study population of *n* = 3,468, 68 samples were a false negative, including the growth of one microbial species (*n* = 24) and the growth of two microbial species (*n* = 3) (Haugum et al., 2021).

There are recent studies that investigated flow cytometry cut-offs that allowed no false-negative rates like in this study. Ippoliti et al. introduced the algorithm based on three parameters generated using the UF-5000 analyzer: WBC, squamous cells (associated with contaminated samples), and conductivity (research parameter related to urine concentration). They suggested cut-off points for squamous cells (>30/μl) or urine conductivity (<6 mS/cm) or WBC (<5/μl) to predict negative samples. These chosen cut-offs were related to sensitivities equal to 100%, specificity of 94%, PPV of 72%, and negative predictive value of 100%. In the total study group (*n* = 1,295), 69 false positives were observed (Ippoliti et al., 2020). A similarly small study group (*n* = 126) was investigated by Song et al. in 2018. The cut-offs for bacterial (50/μl) and yeast-like cells (100/μl) were compared with bacterial and fungal significant growth with no false-negative results. This object focus aims to reduce unnecessary urine culture only by approximately 10% (Song et al., 2018).

Gilboe et al., using the fluorescent flow cytometry (FFC) method, wanted to check the possibility of rapid identification of culture-positive, culture-negative, and contaminated samples as well. Correspondingly, the Norwegian guideline of significant values for different uropathogens in different patient groups were used to define significant bacteriuria—mostly 10^3^ for symptomatic women, children, and men. This study has shown the usefulness of cut-off values from 100,000 bacteria/ml and 10 WBCs/µl. Finally, the assessment led to claim that 95.5% of samples with significant growth from FFC contained bacteria >100,000/ml. WBC ≥10/μl was detected in 93.9% of samples with significant growth but ≥40 WBC/μl only in 60.7%. The prediction of nonsignificant bacterial growth based on the foregoing values permitted reporting 42.1% of samples as negative on the day of sample reception, which was similar to MIM’s results (37.7%) (Oyaert et al., 2018). A higher screened ratio (50.9%) with 99.5% of NPV was achieved by using only the BACT count (cut-off, 15/ul) during the study of Kim et al. in 2018. Kim et al.’s study also delivered information from a specific subgroup of immunocompromised patients (*n* = 399). Different cut-offs were evaluated: the highest had ≥267 bacteria/µl, with sensitivity of 90% and specificity of 93%, whereas the lowest one had ≥27 bacteria//µl (sensitivity of 99% and specificity of 77%), but there were no significant differences compared to the remaining patients’ results (Kim et al., 2018). Haugum K et al., in 2021, obtained 36% culture reduction in the elderly emergency department unit with the cut-off 10^8^ CBU/L (Pieretti et al., 2010). Furthermore, García-Coca et al., in 2017, even increased this rate to 48.2% culture reduction in a case with a representative group (*n* = 17,483) (Alenkaer et al., 2021).

A second Norwegian study evaluated the results from a similar study group (*n* = 1,119) but focused more on elderly patients from an emergency department (*n* = 544) and investigated specifically the patients’ and department unit’s cut-off value (10^8^ CBU/L). This cut-off resulted in 92% NPV (Alenkaer et al., 2021).

However, currently, there is no published meta-analysis for UF-5000/4000, but a comprehensive study of the previous UF-analyzer is available and can support this practice. The meta-analysis for ruling out the negative samples by the criteria based on the UF-100 results manifested good pooled sensitivity values for WBC and BACT at 0.87 and 0.92, respectively (Previtali et al., 2017). A subsequent study associated with results from the previous generation of flow cytometry analysers (UF-1000i) with a large number of samples (*n* = 17,483) used concurrent criteria to exclude negative samples before the culture (>25 WBC/μl or >385 bacteria/μl). The authors describe the categories for microbiology results as follows—negative: <10^4^ CFU/ml and positive: 10^4^–10^5^ CFU/ml and >10^5^ CFU/ml (García-Coca et al., 2017).

Owing to these reasons, we decided to adjust the suggested MIM laboratory cut-offs chosen with “or” instead of “and” criteria without specific cut-offs for male and female.

There are studies intended to check if Iris iQ^®^200 ELITE can be used as a fast clinical screening procedure to rule out clinically relevant UTIs and assess their impact on reducing urine cultures with no growth or nonsignificant growth (Parta et al., 2013; Lee et al., 2019). Stürenburg E et al., in 2014, compared IRIS with culture plating (significant bacteriuria ≥10^4^ CFU/ml). They introduced the way of screening based on mathematical algorithms that contain WBC and bacterial information and compared it with dipstick analysis. The analyzer, instead of quantitative bacterial counts, provided ASP values. The authors have shown a few variants of algorithms, considering indicators that are alone or combined. The best sensitivity of 95%, specificity of 61%, and reduction in urine cultures of 35% were achieved when leukocytes and bacteria and ASP were used in the calculation formula (Stürenburg et al., 2014). Lee et al., in 2019, also used similar parameters (exemplary cut-offs; WBC ≥4/HPF, WBC ≥6/HPF, and ASP ≥8500/μl). The researchers concluded that, for 10^5^ CFU/ml, bacteriuria prediction in culture by Iris iQ^®^200 showed good sensitivity (91.5–92.3%), specificity (49.8–52.6%), and NPV (97.7–97.9) ranges depending on the set cut-off values. On the opposite, when significant bacteriuria was defined as 10^4^ CFU/ml, the screening efficiency was unsatisfactory. The researchers also observed that mainly Enterococcus, among all uropathogens was problematic to predict as positive by using screening methods (García-Coca et al., 2017). These observations are comparable to the study of García-Coca et al. in 2017, where they investigated samples with Enterrococus having lower WBC than the negative samples (Alenkaer et al., 2021).

These two studies were directly comparing UF-5000/4000 and Iris iQ^®^200. The first mentioned study was intended to assess diagnostic performance for urinalysis purposes. Cho J et al. compared five urine sediment analysers including iQ200SPRINT and UF-5000. Manual microscopy using KOVA chambers were treated as a reference method. The numeric data for WBC from both systems were compared. Importantly, for WBC, UF-5000 showed bias within ±20%, but that of iQ200SPRIN was higher (Cho et al., 2019). The second also study makes a comparison for five different systems but, compared the results with cultivation strictly for enhancing the diagnostic efficiency in UTI. The authors adjusted the cut-off values separately for male and female patients. The conclusion from this study was that UF-5000 achieved the best efficiency in UTI screening (the highest area under the curve values) (Cho et al., 2021).

Many authors pointed out that the urinalysis results are quickly available compared to those of urine culture. The turnaround time and interpretation of even complex algorithms are now comparable to those of initial test strip screening. This way of automated screening enables the exclusion of UTI when the presence of inflammation indicators is in a small amount within 1 to 2hrs. At the same time, we have the opportunity to start further tests and differential diagnosis without the need for routine microbiological diagnostics, which lasts at least 24hrs. Gilboe et al., in their study continuation, combined flow cytometry screening to rule out negative samples with direct antibiotic susceptibility testing. This publication presented a detailed chart with a developed workflow, which shows that the distribution rate depends on days. Finally, only 4.1% of all results was reported after 3 days, and 42.1% was reported as negative on the day of sample reception. Furthermore, 53.8% samples were proceeded based on BACT-info (Oyaert et al., 2018). In case of UTI suspicion, automated typing of bacteria may be beneficial for an early decision about the way of treatment or deeper diagnosis. De Rosa R et al., in 2018, next to the UF-5000 potential to rule out UTI samples, also investigated its flagging system to predict the presence of Gram-negative bacteria in samples requested for culture plating. They concluded that the “Gram Neg?” flag predicted Gram-negative samples in culture with sensitivity of 81.6% and specificity of 93.3% (De Rosa et al., 2018).

## Conclusion

Accurate urinalysis provides information on bacterial and leukocyte counts as useful indicators in UTIs, as confirmed by many studies around the world. It could be a future tool for linking clinical and microbiology laboratories. However, cut-off adjustments require individual optimization.

## Data availability statement

The raw data supporting the conclusions of this article will be made available by the authors without undue reservation.

## Ethics statement

The studies involving human participants were reviewed and approved by the ethics committee of MIM with protocol number 17/WIM/2019 (Military Institute of Medicine, 128 Szaserow Str., 04-11 141 Warsaw, Poland). Written informed consent from the participants’ legal guardian/next-of-kin was not required for this study in accordance with the national legislation and the institutional requirements.

## Author contributions

MS contributed to the conception and design of the manuscript and collection of the patient database and wrote the first draft of the manuscript. ZT-O contributed to the review and selection of the literature for the manuscript and participated in the selection of statistical methodology and statistical analysis of the results. MM contributed to the review of the manuscript and participated in the selection of the literature for the manuscript. AW-K contributed to the conception and design of the manuscript and made the final revision of the manuscript. All authors contributed to the article and approved the submitted version.

## Conflict of interest

Author MS is employed by Sysmex Poland Ltd. and privately is a Ph.D. researcher at the Department of Laboratory Diagnostics, Military Institute of Medicine, Warsaw, Poland.

The remaining authors declare that the research was conducted in the absence of any commercial or financial relationships that could be construed as a potential conflict of interest.

## Publisher’s note

All claims expressed in this article are solely those of the authors and do not necessarily represent those of their affiliated organizations, or those of the publisher, the editors and the reviewers. Any product that may be evaluated in this article, or claim that may be made by its manufacturer, is not guaranteed or endorsed by the publisher.

## Abbreviations

UTI, urinary tract infections; MIM, Military Institute of Medicine in Warsaw; AMR, antimicrobial resistance; FFC, fluorescent flow cytometry; EUA, The European Association of Urology; WHO, World Health Organization; LE, leukocyte esterase; NIT, nitrite; HPF, high-power field; TP, true positive; FP, false positive; TN, true negative; FN, false positive; NPV, negative predictive value; PPV, positive predictive value.
